# Independent and Collaborative Contributions of the Cerebral Hemispheres to Emotional Processing

**DOI:** 10.3389/fnhum.2014.00230

**Published:** 2014-04-22

**Authors:** Elizabeth R. Shobe

**Affiliations:** ^1^Department of Psychology, The Richard Stockton College of New Jersey, Galloway, NJ, USA

**Keywords:** hemisphere, emotion, brain, valence, emotion regulation, subcortical, cortical, handedness

## Abstract

Presented is a model suggesting that the right hemisphere (RH) directly mediates the identification and comprehension of positive and negative emotional stimuli, whereas the left hemisphere (LH) contributes to higher level processing of emotional information that has been shared via the corpus callosum. RH subcortical connections provide initial processing of emotional stimuli, and their innervation to cortical structures provides a secondary pathway by which the hemispheres process emotional information more fully. It is suggested that the LH contribution to emotion processing is in emotional regulation, social well-being, and adaptation, and transforming the RH emotional experience into propositional and verbal codes. Lastly, it is proposed that the LH has little ability at the level of emotion identification, having a default positive bias and no ability to identify a stimulus as negative. Instead, the LH must rely on the transfer of emotional information from the RH to engage higher-order emotional processing. As such, either hemisphere can identify positive emotions, but they must collaborate for complete processing of negative emotions. Evidence presented draws from behavioral, neurological, and clinical research, including discussions of subcortical and cortical pathways, callosal agenesis, commissurotomy, emotion regulation, mood disorders, interpersonal interaction, language, and handedness. Directions for future research are offered.

While the debate over hemispheric asymmetries for emotion perception and identification has been ongoing for over four decades, the most recent observations are a mixture of findings such as a right hemisphere (RH) superiority for negative emotions (Kumar and Srinivasan, 2011; Önal-Hartmann et al., 2012; Sedda et al., 2013), a RH superiority for both positive and negative emotions (Hagemann et al., 2005; Killgore and Yurgelun-Todd, 2007; Alves et al., 2009; Bourne, 2010; Cheng et al., 2012; Irish et al., 2013; Najt et al., 2013; Yuvaraj et al., 2013), no asymmetries for positive emotions (Tomarken et al., 1990; Smith and Bulman-Fleming, 2005; Kumar and Srinivasan, 2011; Zhang et al., 2011; Önal-Hartmann et al., 2012; Najt et al., 2013; Sedda et al., 2013), and no left hemisphere (LH) differentiation between emotional and neutral faces (Najt et al., 2013). A recent meta-analysis concluded that the RH processes both positive and negative emotion, but the LH may only process positive emotions (Abbott et al., 2013). Given the evidence to date, it appears that the RH is integral to processing all basic emotions (positive and negative) and is the seat of subjective affect (feeling). However, inconsistent and contradictory findings make the contributions of the LH to emotional processing highly debatable. It is most interesting that regardless of the methodology and populations studied, the RH consistently demonstrates emotional competence, but the LH sometimes does and sometimes does not. Even when couched in terms of approach and avoidance/withdrawal motivations (e.g., Kinsbourne, 1978; Davidson, 1992) to account for findings that anger (a negative motion) and happy judgments have both been associated with the LH (Harmon-Jones et al., 2013), findings are still mixed with regards to the LH (e.g., Alves et al., 2009; Brüne et al., 2013; Fetterman et al., 2013; Najt et al., 2013; see Miller et al., 2013a). When LH competency is observed, it is consistent only in the identification of positive emotions. However, a broader range of evidence suggests that the LH may play a more crucial role in emotional processing at levels beyond simple identification of emotionality that have yet to be extensively explored. The current paper presents a framework that unifies evidence to date from multiple domains including neuroscience, experimental psychology, clinical psychology, and evolutionary psychobiology.

Presented here is a framework that accounts for inconsistencies of findings by suggesting that the involvement of each hemisphere is qualitatively different, occurring at different points or levels in processing of emotional stimuli, making very different contributions to emotional perception and experience, and for very different purposes. The evidence suggests that the RH directly comprehends and processes the emotional valence of stimuli and then generates the affect (feeling) that is consistent with its interpretation. However, the LH does not appear to engage indirect and genuine comprehension of emotional stimuli, nor does it seem to influence subjective affect that is consistent with the specific stimulus. Rather, being proposed is that the LH contributes an additional or secondary interpretation, based on information received from the RH. This secondary interpretation of the LH appears to be positively biased, making an important contribution to regulation of negative emotion and social interaction, both of which are important for planning, decision-making, and self-preservation. Also, being proposed is that the LH maintains a propositional understanding of RH emotional information and enables verbalization that is more informational than experiential. As such, the LH does not seem to be integral to initial perceptual and experiential stages of emotional processing, but seems more attune to the outcomes of RH interpretation and its application to executive functioning, social well-being, and knowledge representation. Thus, the LH may direct our conscious interpretation of and interaction with stimuli, but does so based on the RH direct and genuine interpretation of the stimulus. This further suggests that complex processing of and responding to emotions require a cross-hemispheric collaboration that originates in the RH, and this is particularly true for negative emotions. Suggestions for testing these hypotheses are presented in Section “Future Directions” below.

## Lateral Subcortical and Bilateral Cortical Emotion Networks

The subcortical and cortical networks provide one indication that the RH is the primary interpreter of emotional stimuli and generator of subjective affect, the outcome of which is then shared with the LH for secondary interpretation and modulation. While several recent papers on neural structures underlying emotional processing focus specifically on prefrontal cortices and the interplay between emotion, motivation, and decision making (e.g., Spielberg et al., 2013), the current framework addresses emotional processing that occurs at a more basic level that begins, appropriately, with subcortical networks and their influence on cortical activity. It appears as though the RH is critical for setting in motion one neural pathway that prepares the organism for immediate action and a second pathway that enables cross-colossal transfer, both pathways originating in subcortical structures. Subcortical connections subserve automatic or unconscious processing of emotional stimuli and subsequent activation for physiological preparation. Gainotti (2012) provided extensive evidence for the automatic and unconscious processing of emotion as being mediated by the phylogenetically old RH amygdala, pulvinar, and superior colliculus. The subcortical amygdala–pulvinar–superior colliculus pathway is considered to be a fast and coarse processor of facial stimuli (facial expressions are the most frequently used emotional stimuli) for orienting to and initiating the physiological arousal that accompanies emotion (Liddell et al., 2005, for review also see Johnson, 2005; Tamietto and de Gelder, 2010). The amygdala has long been associated with perception of emotion, specifically faces (e.g., Adolphs and Tranel, 2003), and the arousing effects of emotion (LeDoux, 1996, 2007; Wallentin et al., 2011). Pulvinar, located in the posterior thalamus, conveys salient emotional information quickly to the amygdala (Morris et al., 1999), but also receives from and influences cortical processes (Sherman and Guillery, 1996). Superior colliculus is a midbrain visual structure important for orienting head and eye movements toward a visual stimulus. Retinal fiber innervation to superior colliculus is commonly understood to enable unconscious visual abilities observed in blind-sight patients. Consistent with Gainotti’s (2012) synopsis, subcortical structures of the RH seem most critical for initiating the physiological arousal associated with emotion. For example, Wittling et al. (1998) observed significantly higher myocardium activity following RH as compared to LH viewing of emotional film clips. Further, Spence et al. (1996) demonstrated that physiological responses to emotional stimuli resulted from RH, not LH presentation. As such, the RH amygdala–pulvinar–colliculus pathway automatically, and without consciousness, appears to process basic emotional stimuli and initiate the accompanying physiological responses. Troiani and Schultz (2013) recently demonstrated that unconscious processing of fearful (i.e., negative) stimuli result in a pattern of activity that includes the right amygdala, the right pulvinar, and left inferior parietal cortex, further implicating early subcortical RH involvement followed by LH involvement. Of course, the nature of LH involvement is unresolvable from that study, but it supports the notion of a second cortical–subcortical pathway for emotion processing.

The second pathway, the cortico-pulvinar–cortical pathway may provide the preliminary link between the immediate fast and coarse subcortical processing and the cortical processing of emotion. This may be the precursor for a cross-callosal transfer of emotional information from the RH to the LH. The pulvinar nucleus of the thalamus is directly connected to V4 (extrastriate cortex, part of the “what” system/ventral stream), inferior temporal cortex (TE), and the temporo-occipital area (TEO) (Baleydier and Morel, 1992; Shipp, 2003), and also to posterior parietal cortex (Raczkowski and Diamond, 1980; Webster et al., 1993; Behrens et al., 2003), medial prefrontal cortex (Romanski et al., 1998; Behrens et al., 2003), superior temporal gyrus (Eidelberg and Galaburda, 1982), and cingulate gyrus (Romanski et al., 1998). While there are no connecting fibers between the left and right pulvinar, there are numerous fibers crossing between the hemispheres via the corpus callosum (CC) from cortical areas innervating pulvinar, and several of these have been repeatedly implicated as part of an emotional processing network (Habel et al., 2005; Matsunaga et al., 2009; Park et al., 2010; Kret et al., 2011). For example, van den Heuvel et al. (2009) and Damoiseaux et al. (2006) investigated resting-state networks (RSN) to determine the synchronization of neural structures during baseline activity. The study of RSN contributes to our understanding of neural networks because the associated structures also tend to be functionally networked (Greicius et al., 2009; van den Heuvel and Hulshoff Pol, 2010). van den Heuvel et al. (2009) observed nine different, overlapping RSNs, but most relevant here is the default-mode network (DMN) that links the CC to the medial frontal cortex, the cingulum (white matter extending from cingulate cortex to medial temporal lobe) to medial frontal cortex and cingulate cortex, and the fronto-occipital fasciculi that link the medial frontal cortex, cingulate cortex, and inferior parietal lobes. Other structures belonging to the DMN include posterior parietal cortex (includes inferior parietal lobes) and superior temporal gyrus (Greicius et al., 2004; Fox and Raichle, 2007). Given these associated structures, it is not surprising that the DMN has been associated with integrating cognition and emotion (Greicius et al., 2004), regulating emotion (Grimm et al., 2008; Wiebking et al., 2011), and identifying emotional valence (Sreenivas et al., 2012).

Additionally, because the DMN consists of primarily midline structures, including the CC (the major pathway for inter-hemispheric communication) and cingulate gyrus, whose axons are the genesis of the CC (Koester and O’Leary, 1994; Rash and Richards, 2001), and is functionally associated with different aspects of emotional processing, it follows that a large amount of emotion would be communicated across the hemispheres. Indeed, a high degree of bilateral activity has been observed among DMN structures (Saenger et al., 2012). In particular, Nummenmaa et al. (2012) observed a high amount of synchrony between the structures of the DMN and other emotional processing structures (thalamus, ventral striatum, and insula) during the viewing and appraisal of negatively valenced film clips. Vecchio et al. (2013) also analyzed EEG coherence, concluding that information about negative emotions is transferred between the hemispheres, but that positive emotions were not. These findings provide additional evidence for a link between thalamic and cortical structures during emotional processing and also suggest that information about negatively valenced stimuli in particular is shared between hemispheres. For current purposes, the significance of pulvinar is its connections to both the RH unconscious processing of emotion pathway and to DMN and other emotion relevant cortical structures. Further, because pulvinar consists of association nuclei, operating in both feed-forward (to cortical) and feedback (from cortical) channels, it has been implicated as the major synchronizer of cortical areas. One function may be to synchronize the cortical structures of the DMN during processing of emotional stimuli.

Of interest then, is the type of information pulvinar shares with the cortical areas of the DMN. Pulvinar coordinates cortical function by synchronizing (strengthening communication) various and widespread cortical areas including temporal, occipital, and parietal lobes (Shipp, 2003). For example, during visual selective attention, the synchrony between pulvinar and V4 and TEO is strong, but the synchrony between the two cortical areas alone is weak (Saalmann et al., 2012). Purushothaman et al. (2012) demonstrated that pulvinar also enhances processing in V1 and that removal of pulvinar nuclei prevents visual information from V1 from spreading to other cortical areas. Although pulvinar is divided into several sections (lateral, medial, inferior, dorsal, ventral), it is generally important for synchronizing cortical areas for arousal (Shipp, 2003), selecting attention, and maintaining attentional priorities (LaBerge and Buchsbaum, 1990; Karnath et al., 2002; Ivanov et al., 2010; Li et al., 2012; Saalmann et al., 2012). Grieve et al. (2000) and Pessoa and Adolphs (2010) suggest that pulvinar is especially important in orienting individuals toward biologically important stimuli. Consistent with these findings, Padmala et al. (2010) suggest that the cortico-pulvinar–cortical pathway is important for directing attention to emotional stimuli that may have a weak signal, but are nonetheless biologically significant, such as faces. Nguyen et al. (2013) observed that pulvinar neurons do selectively respond to facial stimuli at short latencies (50 ms), and then engage in rudimentary categorization of stimuli (faces, face-like, eye-like, simple geometric) at longer latencies (100 ms) (Maior et al., 2010).

The RH amygdala–pulvinar–superior colliculus together with the cortico-pulvinar-cortico connections provide neural pathways by which emotional stimuli are subcortically processed by the RH and then cortically processed via pulvinar’s widespread synchronization of cortical areas, perhaps separating biologically significant emotional stimuli from other stimuli. Johnson (2005) proposed that the subcortical route that is present in infants also serves to activate cortical structures that are important for expression and comprehension of social aspects of emotion. The pulvinar nuclei may be the key structure in the mechanism by which subcortical processing of emotional stimuli becomes cortical by elevating cortical attention to specific emotionally relevant stimuli. It is also worth mentioning that the anterior thalamus has long been associated with mood or subjective affect due to its numerous connections to limbic structures (Yakovlev et al., 1960; Taber et al., 2004). As such, the anterior thalamic nuclei’s contributions to subjective affect coupled with pulvinar’s contribution to processing emotional stimuli may converge in the cortical areas (particularly in cingulate cortex, Shackman et al., 2011), as these emotional aspects seem somewhat independent at the level of thalamus.

## RH and LH Disconnection

The preponderance of studies on hemispheric asymmetries for processing emotions and subjective affect (feelings) have utilized presentation of facial expression stimuli, consciously perceived and labeled by participants. By recording response times, accuracies, and neural correlates to the identification of emotional expression, researchers have commonly observed RH competencies (often superiorities) for all emotional expression, and some observe LH ability to identify positive emotions, only. This methodology reveals cerebral asymmetries for perceiving and labeling emotional stimuli, but provides little information about the kind or depth of processing that is done by each hemisphere or how the hemispheres collaborate for normal emotional processing. Studies on the processing abilities and subjective affect of patients with corpus callossal abnormality (i.e., agenesis, commissurotomy) and with unilateral damage provide insight into the separate competencies of the hemispheres and evidence that normal emotional experience requires inter-hemispheric collaboration. The findings from these populations also suggest that the RH is the true seat of emotional experience, attuned to on-line processing (comprehension, identification) of specific emotional stimuli and also creating stimulus appropriate subjective affect. The LH, on the other hand, appears to be disconnected from the on-line, direct processing of emotion, relying on the RH for the transfer of such information and making abstract, propositional extractions. When the LH must process or interpret emotional stimuli in the absence of a RH contribution, the LH appears to be disconnected and lost, resulting in either confabulation or a default positive response bias. This is consistent with Gazzaniga’s (1998) notion of the LH acting as interpreter and with the observations of commissurotomy patient V.P. discussed by Gazzaniga and LeDoux (1978). In V.P.’s case, his RH was able to correctly interpret a frightening scene, which resulted in a sufficient amount of physiological arousal. Interestingly, V.P.’s verbal LH appeared to have successfully interpreted the physiological arousal but was completely unaware of its origin, and therefore, confabulated a verbal reason (e.g., “the experimenters and/or room were unnerving”). V.P.’s severed CC resulted in the inability of the LH to directly interpret the emotional stimulus, forcing a reliance on other cues and the resulting confabulation. V.P. illustrates the interpretive nature of the LH or at the very least, that the LH does make interpretations of circumstantial cues in the absence of true comprehension of the causal stimulus. In their review of V.P. and several other findings, Baynes and Gazzaniga (2000) similarly (originally) conclude that the RH makes the situation-appropriate emotional contribution to the LH interpretation of events. Cleverly, they also propose that if this were true, it should be possible to “trick” the LH. This point will be returned to in the Section “Future Directions” below.

In addition to V.P., other commissurotomy studies demonstrate that the speaking LH appears to have lost access to the understanding of the emotional nature of a stimulus. For example, Bermond (1995) observed that CC dysfunction results in deficits in verbal descriptions of emotion, but intact experiencing of basic emotions. Classically, commissurotomized patients are able to use non-verbal indications that they comprehend emotion, but are unable to verbalize these feelings (Sperry et al., 1979). Commissurotomized patients also tend to use left-sided gestures to communicate emotion (i.e., shrugs, batons) (Lausberg et al., 2007), indicating a RH dominance for spontaneous emotional communication. Moreover, the deficits of CC patients do not seem to be a general inability to accurately understand or communicate emotions. For example, Benowitz et al. (1983) observed intact identification of facial emotions presented to the RH, but poor performance when presented to the LH of commissurotomized patients. As such, the problems of CC patients are in their verbal descriptions and the inability of just the LH to understand and communicate emotions.

Similarly, others have observed that a breakdown in inter-hemispheric communication via the CC in the absence of unilateral damage results in a LH that seems unable to effectively process emotional stimuli, understand the relationship between events and appropriate affect, and apply affect during discourse. For example, Paul et al. (2006) observed that ratings of facial-emotion valence (happy/sad) and degree of arousal (excited/calm) by patients with agenesis of the corpus callosum (AgCC) were highly inconsistent (many completely inaccurate), observing particular difficulty with negative emotions. AgCC also tended to provide lower than normal assessments of arousal. Interestingly, their GSRs (measurements of physiological arousal) were normal, suggesting that the RH subcortical pathway was normally functioning, and the absence of RH damage assumes this as well. Consistent with V.P., it appears as though the verbal LH may have been interpreting that there was some kind of emotion, perhaps based on physiological arousal, but in the absence of accurate information from the RH, it was unable to decipher the precise quality of the emotion. Additionally, O’Brien (1994) observed that AgCC older children showed normal use of language (e.g., vocabulary, sentence structure), but overuse of clichés, deficits in comprehension of jokes, social cues, or facial expressions, and speech that was frequently irrelevant or out of place. This suggests that without the RH, the LH is also impaired in the correct application of emotional language.

The disconnection between emotional experience and language is also evident in the content of AgCC speech. For example, Turk et al. (2010) observed significantly diminished use of emotional words in Thematic Apperception Test descriptions. Buchanan et al. (1980) also describe an AgCC patient as having difficulty with labeling emotional speech and prosody, and verbalizing feelings. Paul et al. (2003) also observed that AgCC patients exhibited significant impairments at matching prosody with their verbal labels, and higher frequencies of misinterpretations, use of non-emotional concrete words, and fewer words in their verbal explanations of idiomatic phrases. The deficits in idiom verbal descriptions and use during speech observed by Paul et al. (2003) and O’Brien (1994) are particularly interesting because idioms are emotionally laden, describing an emotional state or predicament of the observer or the observee (Pandian, 2012). As such, a true understanding of idioms requires accurate comprehension of situation-specific emotions. Moreover, idiom deficits do not appear to be simply a general cognitive deficit or byproduct of an abnormal brain in the AgCC population. Instead, Brown and Paul (2000) observed a preserved ability to match idioms to multiple choice meanings in conjunction with deficits in their verbal explanations (their stimuli did include some proverbs, but were not analyzed separately). This indicates that the comprehension of AgCC for verbal emotional stimuli is intact, but verbal expression is impaired. Idiom comprehension is a RH function, where RH-damaged patients show severe deficits in spoken and multiple choice descriptions (e.g., van Lancker and Kempler, 1987; Brownell et al., 1990). Brown and Paul’s findings suggest that preserved idiom comprehension of AgCC was likely due to the intact processing of the RH, the outcomes of which could not transfer to the LH for verbal responding (for review of RH language abilities, see Beeman and Chiarello, 1998; and Lindell, 2006). Further, because idioms are learned by their use in context (Cain et al., 2009), they may be particularly well-suited for acquisition and semantic processing by the RH. Not only does the RH appear to have the advantage for emotional comprehension, but it also has an advantage for comprehending words in contexts (Ince and Christman, 2002), and making appropriate verbal inferences for emotional text (Tapiero and Fillon, 2007). Given these RH superiorities over the LH, it is not surprising that AgCC understand the context and emotion of idioms, but provide inaccurate verbal description. Indeed, children as young as 5 years old use context to derive meaning from idioms, and this is not dependent upon verbal skills; whereas 7-year-olds show accurate knowledge of idiom meaning and this is related to language skills (Le Sourn-Bissaoui et al., 2012). This suggests that the strong ties to language for well-learned clichés may be responsible for their spontaneous use by AgCC patients, and the inappropriateness or out of place use in spoken language appears to be due to the LH being disconnected from the RH emotional competence. In essence, the LH by itself appears to have no access to the emotional aspects of language, just the linguistic and semantic components.

However, the picture is more complex than assuming that commissurotomy or AgCC creates a RH that is simply “locked in.” That is, a truly normal emotional experience appears to require some cross-hemispheric communication. Notably, commissurotomized patients are also likely to show some alexithymia (deficits with experiencing and processing emotion) (Hoppe and Bogen, 1977), suggesting that their non-verbal emotional experience is not entirely normal. Further, alexithymics show less frontal coherence between the RH and LH, indicating less information transfer from the RH to the LH (Houtveen et al., 1997). This could be due to the inverse relationship between size of the CC and severity of alexithymia (Habib et al., 2003). Lumley and Sielky (2000) observed similar findings of an association between alexithymia and poor inter-hemispheric transfer in normal participants, as did Romei et al. (2008) and Parker et al. (1999). Importantly, alexithymia does not seem to be due to hypoactivity of the RH, rather the RH shows significant activity for both positive and negative emotions (Aftanas and Varlamov, 2004, 2007). Taken together, the observations of commissurotomized, alexithymia, and AgCC patients indicate that normal emotional functioning requires cross-callosal communication. Specifically, it appears as though there is a disconnection between the RH propensity to accurately comprehend emotional stimuli and generate appropriate affect with that of the LH to verbalize and apply emotional information during social and communicative interaction, and perhaps also to add emotion to the conscious experience (as Gazzaniga and LeDoux proposed).

## LH Contribution to Emotional Experience

The empirical literature strongly supports the notion that the RH is integral to the processing of all basic emotions and is especially important for the processing of negative emotions (also known as the RH hypothesis) (Abbott et al., 2013). While several researchers have also observed hemispheric asymmetries for valence (RH, negative; LH, positive) (Davidson and Fox, 1982; Davidson, 1992, 1993, 2004; Davidson and Hugdahl, 1995), I could not locate a single paper published in recent years that showed clear support for this hypothesis. The closest support for the valence hypothesis is from Jansari et al. (2011), who used a fee-viewing chimeric face task. While their findings clearly indicate that positive emotions are better identified from the right side of the face, and negative from the left side, the chimeric face task is not a strong test of laterality owing to the RH ability to tap into both left and right sides of space. This is further clouded by Jansari et al.’s (2000) earlier finding that only females show the valence effect, whereas the Jansari et al. (2011) reported no gender × valence interaction for accuracy. Even though the RH hypothesis has received the most support, there is enough inconsistency to suggest that the LH plays some role in emotion processing. The nature of that role has not been systematically investigated to date nor have any clear theories been put forth, probably because most studies investigating hemispheric emotion asymmetries have been designed for detection and labeling of stimuli, not for gaining insight into how or the depth by which this is accomplished by the hemispheres. Certainly, at this point, we know that the LH can identify positive emotions, but evidence suggests that the LH comprehension of emotional stimuli is shallow, at best. That is, the previous discussion lends to the conclusion that the LH relies on the RH for early identification of the emotional nature of stimuli, and where that is not possible, the LH is inaccurate (positively biased) and confabulatory. As such, reports of LH advantages for identifying emotional stimuli are perhaps due to implementation of strategies other than initial and genuine comprehension of the emotional nature of a stimulus.

However, beyond the preliminary RH processing of emotion, the LH may be crucial for other aspects of emotion, some of which are dependent upon the RH sharing of information and some that may be independent of stimulus but dependent upon motivation. For example, the LH may be involved with regulating the emotion response of the RH or the LH may unilaterally induce a positive mood regardless of the situation. In either case, the LH may be more involved with incorporating emotions into higher cognitive functions to produce refinements or alterations to behaviors that would be otherwise driven purely by subjective affect. To use an analogy, the RH acts as the soldier in the trenches who responds to the onslaught of battleground stimuli, interpreting friend from foe, while the LH acts as higher level commanders, receiving but not directly experiencing information from the frontline, strategizing how to gain an advantage or attain peace, each of which requires keeping a level head and maybe a bluff.

One contribution made by the LH may be toward regulating emotions. Emotional regulation is the implementation of strategies to increase or decrease the intensity of emotional experience (Gross, 2002). Because emotions motivate us to approach (e.g., joy, love), avoid (e.g., sad, disgust), or attack (e.g., anger, disgust) (Stosny, 2011), their regulation lowers stress reactivity, enhances social well-being (i.e., preventing a “knee-jerk response”), and enables attainment of instrumental rewards (e.g., smile politely at a horrible boss to get a raise). The LH does appear to be much less emotionally intense than the RH. For example, participants with greater RH activity reported more intense positive and negative feelings after viewing film clips, but those with higher LH activity showed less intense feelings (Hagemann et al., 2005). Similarly, observations of two commissurotomized patients by Schiffer et al. (1998) also suggest that the LH experiences emotions much less intensely than does the RH. This attenuation of emotional intensity maybe particularly practical in the regulation of negative emotions (Eippert et al., 2007). For example, the LH shows superiority over the RH for overcoming emotional distractors (Dolcos et al., 2006), is preferentially activated during emotional regulation caused by unpleasant stimuli (Davidson, 2004; Parvaz et al., 2012), and characteristic LH activation asymmetry results in more adaptive cardiovascular responses to social threats (i.e., negative feedback) (Koslov et al., 2011) and also attenuated startle responses following offset of, but not during negative stimuli (Jackson et al., 2003). Further, LH frontal gyri abnormality (abnormal thickness) is related to deficits in emotional regulation (Wilde et al., 2012). Additionally, attenuation of an emotional response does not appear to be a general LH “numbness,” but may instead be a capitalization on LH processing strategies. The most effective emotional regulation strategies are reappraisal and problem solving (Aldao et al., 2010). Reappraisal, or altering distress by reinterpreting events as positive or benign (Gross, 1998) has been associated with LH prefrontal activity (Ochsner and Gross, 2004; Kim et al., 2012). Problem-solving that requires significant planning to arrive at a solution, such as the Tower of London, has also been associated with LH frontal (Morris et al., 1993; Owen et al., 1996) or bilateral activity (Baker et al., 1996) in the medial, inferior frontal, and cingulate gyri, and the LH supramarginal and angular gyri (Lazeron et al., 2000). Reappraisal and problem-solving strategies also involve a significant amount of verbalization (e.g., self-talk), and several of the LH structures activated during these activities are related to language. Nonetheless, the LH role in emotion regulation also suggests that it is more involved with using and altering emotions than directly perceiving and identifying the emotional nature of stimuli.

Heilman and Bowers (1990) also proposed an inhibitory role for the LH that is akin to emotional regulation. They suggest that LH dysfunction results in a disinhibition of the RH, which in turn, increases physiological arousal associated with emotion. Although their proposal was conjecture at the time of their writing, these more recent findings lend additional plausibility. One additional benefit to emotional regulation may be to maintain a coherent and consistent conscious experience, for which Ramachandran (1995) has suggested the LH to also be dominant. This enables allocation of attention and other cognitive resources to tasks and goals extending beyond the immediate circumstance. Greenberg (2007) also argues that the LH mediates emotion regulation by altering conscious thoughts or interpretations of stimuli. The strategies reappraisal and problem solving most certainly involve an alteration of conscious interpretations. Interestingly, Banks et al. (2012) observed greater GSR in the left-hand (RH contralateral) to implicit presentation of positive and negative facial expressions, but greater right hand (LH) GSR to explicit presentations of negative expressions. This implicates the early role of the RH in emotion processing and the importance of LH involvement during conscious stages of emotion, but also that the LH involvement at more advanced levels may be restricted to additional cognitive processing of negative emotions.

Observations of clinical populations with affective disorders provide additional clues that the LH is important for regulation of negative emotions. Panic disorder (PD) characterized by fear, anxiety, and avoidance has been associated with hyperactivity of the RH (Drayer et al., 1989; Smeets and Merckelbach, 1997; Wiedemann et al., 1999). PD patients express fear or anxiety normally, but these emotions are invoked too frequently (such as baseline or rest phases, Gorman et al., 1988; Wilhelm et al., 2001) and show an attentional bias for threatening information (McNally et al., 1990), which is also suggestive of a hyperactive RH. Alternatively, Akiyoshi et al. (2003) using NIRS, observed LH hypoactivity, but not RH hyperactivity in PD patients. Interestingly, Prasko et al. (2004) observed that symptom reduction following pharmacological and cognitive-behavior treatment of PD coincided with reduced activity (PET/FDG uptake) in the RH and increased activity in the LH, suggesting that PD may be associated with both LH hypoactivity and RH hyperactivity. Depression has also been associated with LH hypoactivity (Henriques and Davidson, 1991). LH damage can result in reduced motivation and activity (abulia) (Förstl and Sahakian, 1991; Caeiro et al., 2013) and increased apathy (Kang and Kim, 2008; Onoda et al., 2010), each of which are symptomatic of depression. Further, these symptoms prevent attainment of goals, coinciding with a negative emotional state but are not themselves emotional states. However, a dysfunctional LH and resulting absence of emotional regulation would result in an emotional landscape dominated by negative feelings (e.g., Rottenberg, 2005; for review see Hecht, 2010). Indeed, depressed patients have a particular difficulty with emotional regulation (Pietrek et al., 2012). It is unlikely that a hypoactive LH and resulting depression indicates a lack of positive emotion and therefore, valence asymmetries because there is ample evidence demonstrating RH capability for both positive and negative emotion. Deep brain stimulation has been demonstrated to alleviate depression (Lozano et al., 2012) further implicating a pathology of neural hypoactivity. RH hyperactivity that has been observed in depressed patients appears to only be associated with stress, anxiety, and panic symptoms (Hecht, 2010). As such, it is possible that PD patients exhibit both hypoactivity of the LH and hyperactivity of the RH, where depression is associated mostly with LH hypoactivity (Iznak et al., 2011; Arnsten and Rubia, 2012). In both PD and depression, negative emotions are poorly regulated, and the added RH hyperactivity in PD patients appears to also add additional intense and negative emotions. At the least, their behaviors suggest that LH hypoactivity is a common underlying pathology. This is also suggested by the observations that PD is frequently comorbid with depression (Leckman et al., 1983; Gorman and Coplan, 1996; Kessler et al., 1998), and this comorbidity is more likely after LH stroke (Bhogal et al., 2004; Barker-Collo, 2007; for review). In keeping with the evidence suggesting that negative emotions in particular are shared between the hemispheres, the nature of each hemisphere’s involvement may be that the RH mediates the subjective affect and the LH regulates it. In PD and depression, emotion cannot be regulated due to suggested hypoactivity of the LH.

Patients with RH damage (therefore, RH hypoactive) show the opposite affect problems. Specifically, patients with RH damage exhibit mania (Sackeim et al., 1982; Starkstein et al., 1989; Lee et al., 1990; Morris et al., 1996; Paradiso et al., 1999; for review see Santos et al., 2013), and this is much more bizarre and inappropriate than depression or even PD. Manic bipolar patients show decreased RH activity in response to fearful facial expressions (Harmon-Jones et al., 2002; Killgore et al., 2008; Versace et al., 2010), and cortico-vestibular stimulation of the left ear increases RH activation and reduces mania (Blumberg et al., 2000). Early uses of the Wada test (Wada, 1949) also confirm that immobilization of the RH results in mania, whereas the same for the LH results in catastrophic and depressive responses (e.g., Perria et al., 1961). Manic and euthymic bipolar patients are also relatively poor at recognizing facial emotions, as compared to healthy controls (Lembke and Ketter, 2002; Bozikas et al., 2006), further implicating RH dysfunction. If we only considered the affect disordered populations, we could assume valence asymmetries (LH, positive; RH, negative), as did the originators of the valence hypothesis. However, if this were true, then patients with dysfunction of the CC should consistently show happy speech and have no problems with happy valence discourse or facial expressions. But, as previously indicated, that is not the case.

Collectively, observations of collosal and affective clinical populations more plausibly suggest that a LH disconnected from the RH (due to callosal or unilateral damage) results in LH emotional confusion and verbal confabulation. When the two are connected, a damaged LH appears to release or fail to regulate the negative emotions of the RH and a damaged RH provides no information about negative stimuli or feelings to the LH. Pathological mania due to RH dysfunction could therefore, be an outcome of continued up-regulation of mundane non-emotional experiences in the absence of RH input or it could be due to a general positive bias of the LH. The latter suggestion seems more plausible because it is more consistent with previous literature demonstrating that the LH has at least some competence for positive emotion. Further, because the LH appears to exhibit less intense emotions under normal circumstances, a damaged or hypoactive RH forces emotional processing onto the LH, which only seems to be independently capable of positive assessments.

The behaviors of manics and depressives also indicate that the LH is poor at interpreting the emotional nature of stimuli. First, depression or catastrophic reactions would be considered normal responses to an experience of traumatic brain injury, sudden disability, and traumatic life events (a frequent trigger for clinical depression). As such, the depression observed in LH damaged patients is actually more normal and appropriate to their situation suggesting that the RH at least accurately appraises the situation. By comparison, the manic reactions of the LH are truly inappropriate to the experience of brain injury and disability, and suggest that the LH does not have the capability to comprehend and apply the appropriate emotional response. Even in non-TBI populations (unilateral hyper/hypo activity), it appears as though only the RH is in tune to the emotionality of a situation or stimulus, whereas the LH relies on a positive bias that may or may not be situation-appropriate.

Further underscoring the bizarre and out-of-touch emotional behavior of LH-reliant manics is that persistent and overly positive interpretations of stimuli are survival disadvantages. More advantageous is a negative and fearful bias, a bias that is more prominent in the natural behaviors of prey animals because it decreases the chances of being a victim of predation. As such, from an evolutionary perspective, it seems more advantageous to consistently interpret ambiguous or novel stimuli as negative and quite disadvantageous to be persistently positive. The exception to this is the advantage of interpreting kin and some familiar stimuli as positive, but this, too, appears to be a RH function. For example, infant–caregiver emotional bonding coincides with RH, not LH activity (Schore, 2005; Noriuchi et al., 2008; Minagawa-Kawai et al., 2009). Moreover, one common phylogenetical argument for asymmetry is that a dual or lateralized brain enables for greater modularity, and therefore a much wider repertoire of abilities and behaviors. As species evolved, brains evolved with the addition of mass (and hence, structure and function), and so later evolved species maintain structures of earlier species with the added benefit of additional brain. This is mirrored by observations that evolutionarily older structures appear prior to newer structures during prenatal development (“ontogeny recapitulates phylogeny”) (Previc, 1991), and the RH does seem to have evolved earlier than the LH (Gupta et al., 2005; Howard and Reggia, 2007; Carmona et al., 2009). Given the advantages of avoiding predation and attaching to kin, and the earlier development of the RH, it is advantageous for the RH to possess competencies to accurately and appropriately interpret both positive and negative emotional nature of stimuli. Further, the RH may do so with a greater likelihood of negative interpretation for non-kin or non-drive stimuli which, incidentally, encompass the vast majority of stimuli used to study emotion asymmetries, such as facial expressions of complete strangers. It is also reasonable that the evolution of the LH would not add simple redundancy to this process, but would instead, add something valuable or that provides a survival advantage. As such, the LH probably did not take over positive emotion from the RH, but added new processing strategies to the information already sufficiently processed by the RH. These processing strategies may include emotional regulation, but may also be a more general positive response bias. At this point, two questions arise: (1) is the LH positively biased, without directly interpreting the emotional nature of stimuli? and (2) what possible selective advantage could this have?

There is some evidence that the LH has a general positive bias in the absence of true emotional comprehension. Nijboer and Jellema (2012) presented the case of a patient with extensive RH damage who, as expected, had difficulty recognizing most emotions but appeared normal for positive emotions. Upon closer inspection, this patient was simply biased toward providing positive responses, without any true comprehension of the positive expression. This case illustrates that the LH may not directly interpret stimulus emotionality, where observed valence asymmetries reflected a LH response bias. Note then, that typical lateralized half-field presentations of emotional faces cannot detect this bias. Rather, a LH bias would reveal accuracy to be much higher for positive and lower for negative rvf/LH stimuli, and high accuracy for both positive and negative stimuli in the lvf/RH. This is a fairly common finding where authors concede partial support for both the valence and RH hypothesis. Even where differences between emotional and neutral faces are observed in each hemisphere, it is potentially confounded because emotional faces and neutral faces differ on more than one dimension, where neutral expressions are not necessarily part of an emotion continuum because they are the absence of emotion. As such mental processes driving responses to emotional faces may be different from those driving responses to neutral faces (e.g., Bobes et al., 2000). The findings of Nijboer and Jellema (2012) warrant experimental replication, but future researchers could also utilize signal detection analyses for clarification between response bias and accuracy. To my knowledge, there have been few studies applying signal detection analysis to emotion processing tasks. One demonstrated a response bias with happy faces, but greater sensitivity to negative faces in a free-viewing task (Schulz et al., 2007), another demonstrated greater sensitivity for RH presentations of low-intensity positive stimuli (Snodgrass and Harring, 2000), and a third demonstrated greater sensitivity of the RH to emotional pictures and faces (Snodgrass and Harring, 2004). Each of these studies suggests that the LH may not be particularly accurate and maybe biased to give positive responses in the absence of true comprehension of affect. As such, a LH advantage for responding to positive stimuli used as support or partial support for the valence hypothesis is not surprising given that the LH may be consistently and inherently primed to do so.

If the LH truly has a default bias to respond positively or promote positive affect, then this should present as an advantage. One such advantage would be that interpreting events as positive and giving the impression of positive emotion is beneficial to social interaction. People tend to overemphasize positive and overreject negative descriptions of the self (Paulhus and Trapnell, 2008). Kircher et al. (2000) observed a LH dominance during PET recordings of participants making decisions about their own personality traits (replicated by Faust et al., 2004), and the LH is also more likely to endorse likable traits about ourselves (Marsolek et al., 2013). Further, patients with RH damage following stroke present an extreme positive view of their predicament, claiming fewer or outright denying physical deficits (e.g., anosognosia) (Vocat et al., 2010; Azouvi and Peskine, 2013). So, the LH appears to improve our perception of our own shortcomings. The LH may also enable us to perceive others more positively. For example, LH stimulation by rTMS results in non-hostile attributions of other’s intentions (Giardina et al., 2011). Positive emotional responses are also more socially acceptable than negative responses, and people prefer to interact with those who have positive versus negative outlooks, and positive moods induce social interaction (Harker and Keltner, 2001; Lyubomirsky et al., 2005; Whelan and Zelenski, 2012; Miller et al., 2013b). As such, it is in our best social interests to actively engage in impression management by consistently promoting positive aspects of ourselves, showing positive emotional responses, and perceiving others (perhaps via facial expression) as non-hostile and approachable. Importantly, this does not suggest that the LH is the seat of “self-awareness” (Keenan and Gorman, 2007 provide a case for RH as the seat of “self”). Rather, this suggests that the LH is the seat of an idealized self, one that maintains or constructs a consistent, conscious sense of self (as suggested by Ramachandran, 1995), and enables for self-control (as suggested by Greenberg, 2007). The LH then, appears important for managing social impressions and promoting social interaction using a consistently positive frame. Pathological mania is an extreme presentation of this, and as has been previously indicated, results from a dysfunctional RH. This also suggests that while the LH may be disconnected from directly experiencing and processing the emotional nature of stimuli, in a normally functioning brain this has some very tangible benefits. Buck (1999, 2002) and Ross (1997) also advanced the idea that the LH is important for pro-social emotions, arguing in favor of a communicative gene hypothesis where fitness depends on the ability of genes to cooperate and communicate with other genes in the population pool (self or others). As such, it is perhaps advantageous for the LH to be disconnected from the direct and preliminary interpretation of the emotional nature of a stimulus, in favor of facilitating social interaction and goal attainment through regulating negative emotion and positive bias. Extending this, we could further argue that the LH cons or masks the self and others as a means toward goal attainment, making this adaptive.

In keeping with the notion that the LH is important for integrating emotional information received from the RH into higher level cognitions, it is reasonable to suggest that the LH transforms this information into propositional knowledge for categorization and verbalization. In McGilchrist’s (2010) review of the literature on cerebral asymmetries, he cites several studies that demonstrate a LH propensity for categorizing and storing abstract or propositional knowledge, as compared to the RH propensity toward “real-world views” and organization of knowledge by specific exemplars. This aligns with the findings of Kensinger and Choi (2009), where the RH remembers details about emotional events, but gist information is remembered better when it is positive and presented to the LH. Further, while it is generally accepted that the RH mediates prosody, it is often observed that both RH and LH damaged patients have prosody deficits. The specific deficit in RH-damaged patients seems to be comprehension of the emotional state of the speaker (emotional prosody), whereas LH-damaged patients show deficits linking the emotional meaning to propositional speech that include differentiating interrogative, imperative, exclamatory statements (linguistic prosody) (Ross et al., 1997). Witteman et al. (2011) conducted a meta-analysis on LH- and RH-damaged patients to process prosody and concluded that RH patients show deficits in emotional prosody, but LH patients show primarily linguistic prosody deficits. In Abbassi et al. (2011), extensive review of emotional word processing, they provide evidence for LH advantage and/or activity for the initial processing of emotional words, but that a RH advantage emerges shortly thereafter. They conclude that the LH has an advantage for early semantic decoding of emotional words, but that the RH engages later. They also suggest that the RH involvement may be due to the attention-grabbing qualities of emotional stimuli. However, in light of the currently reviewed literature, it seems more likely that the LH quickly decodes the semantic meaning of the word, but the RH mediates the emotional comprehension and subsequent affect. Mirroring the findings with AgCC patients reported above, Sherratt (2007) observed that RH-damaged patients used evaluative words rather than expressions of feelings during discourse on personal topics as compared to controls, and had particularly difficulty with negative compared to positive discourse. These findings suggest that the LH dominates semantic knowledge about emotional words, but may not be able to apply those words to emotion-relevant discourse without the RH sharing its comprehension and subjective affect. The LH role in the linguistic encoding of emotional information is underscored by the observations that brain areas that are activated in the LH during emotion processing tend to be language-related structures (Killgore and Yurgelun-Todd, 2007, described in greater detail, below). This, too, suggests that only the RH is engaged in the direct processing of the emotional nature of stimuli, but also that functional neural modules (e.g., Broca’s area for speech, Wernicke’s for comprehension) are part of larger systems that apply general strategies to information processing. For example, LH language and categorization processing is applied not just to linguistic stimuli, but to other domains, such as emotion. Johnson-Frey et al. (2005) observed LH dominance in activity in distinct frontal, prefrontal, parietal, and temporal regions during planning and execution of tool use. Moreover, this was concurrent with activity in LH semantic regions suggesting that the LH dominance for language processing may also underlie execution and planning for the domain of tool use LH dominance for motor skills was also observed by (Janssen et al., 2011). As such, it is plausible that processing strategies of the LH would be applied to multiple domains.

## Inter- and Intra-Hemispheric Processing of Emotion

Processing positive emotions is easier than negative, as accuracy and response time advantages to positive over negatively valenced stimuli are frequently reported (Hugdahl et al., 1993; Compton et al., 2005; Jansari et al., 2011; Najt et al., 2013). This appears to be due to additive LH and RH processes. If the RH processes all emotion and seems biased to negative emotion, then the automatic assignment of positive labels by the LH adds to the accuracy and response time of the RH for processing of positive emotions, generally. For example, Najt et al. (2013) observed a main effect for positive over negative emotions, but the interaction revealed only a RH advantage for negative and no differences between the hemispheres for positive emotions. Using a different methodology, Compton et al. (2005) presented emotionally expressive (they only used happy and angry) or neutral faces along with a third target face in either visual field. They observed an advantage for inter-hemispheric matching of the target to the samples and lvf/RH advantage for intra-hemisphere presentations. Killgore and Yurgelun-Todd (2007) presented sad and happy faces to the LH and RH, and recorded subsequent fMRI activity. They observed that unilateral presentation of happy faces resulted in activity restricted to the hemisphere of presentation. These findings suggest several points. First, either hemisphere can identify a positive face. Second, the RH is superior to the LH at perceptual matching of positive (happy) and negative (angry) even though both have been characterized as “approach.” Third, when both hemispheres simultaneously contribute to the task, the advantage for identifying emotional over neutral stimuli is even greater than, but not different from just one hemisphere alone. These findings are consistent with Banich and Belger (1990), Belger and Banich (1992, 1998), and Weissman and Banich (2000) proposal that easy tasks (lower processing complexity) benefit from intra-hemisphere processing, but difficult tasks benefit from inter-hemispheric. Compton et al. also argue that their perceptual matching task has additional complexity over identification of a single stimulus. Additional evidence that both hemispheres identify positive emotions comes from observations that individuals with LH damage are not always impaired for positive emotions (Adolphs et al., 1996; Borod et al., 1998, 2002), individuals with LH temporal lobe epilepsy are not impaired at recognizing positive emotions, and intact individuals consistently show a RH/left side bias for identifying happy chimeric faces (Levy et al., 1983; Voyer et al., 2012). Because positive emotions can be identified by either hemisphere, they do not require any inter-hemispheric collaboration, and so main effects for positive over negative words are most likely due to additive, and not interactive, effects of RH and LH abilities.

Conversely, prior research suggests that only the RH can identify, comprehend, and feel negative emotions. I have also suggested, here, that for the LH to work with negative emotion (e.g., regulation, language), this information must be transferred from the RH to the LH. In addition to Killgore and Yurgelun-Todd (2007), finding that identification of positive emotions can be accomplished within either hemisphere, they observed that unilateral presentation of sad faces, resulted in bilateral activity. While the depth of processing accomplished by each hemisphere was not directly explored by Killgore and Yurgelun-Todd, it is important to note that the reported LH activity for processing of both happy and sad faces was restricted to regions that are related to language, such as left inferior and medial frontal gyri (syntax, Tyler et al., 2011), medial temporal gyrus (language and semantic memory, Tranel et al., 1997; Chao et al., 1999; Cabeza and Nyberg, 2000; Ashtari et al., 2004), insula (speech, Dronkers, 1996; verbal emotion, Ardila et al., 1997; Ackermann and Riecker, 2004), frontal cortex, fusiform gyrus (face processing in the RH, phoneme/grapheme in the LH, Démonet et al., 1994; Chance et al., 2012), and “lingual areas.” These findings also support the previously mentioned hypothesis that one role of the LH in emotion processing is for verbal encoding and other language-related, higher cognitions such as planning. It is also noteworthy that some of these structures (frontal gyri) are also part of the bilateral DMN, discussed earlier as being important for processing emotion. As such, the initial involvement of the LH in inter-hemispheric processing of negative emotion appears to be restricted to linguistic contributions.

## Incorporating Valence, RH, and Approach/Avoidance Hypotheses

The idea that only the RH comprehends negative valence is not original to this paper (e.g., Najt et al., 2013), but the evidence presented in this paper is consistent with a RH advantage for identification and comprehension of the emotional nature of stimuli, and also mediates a reactive affective response. This appears to be true regardless of valence and approach/avoidance motivations. Conversely, the LH does not appear to have this same ability for any emotion. The LH appears to have a default positive bias in early stages of processing emotional stimuli, suggesting that it relies on the RH for accurate interpretation of emotional stimuli. Also, consistent with the findings and proposals of others cited herein, the LH plays a major role in more advanced and different aspects of emotion processing (regulation, impression management, and verbal and propositional coding) important for social interaction, language, executive reasoning, and a coherent conscious experience. Importantly, this does not necessarily propose new functions for the LH, just an application of LH dominant processes to emotional information. Additionally, this framework incorporates elements from the three prevailing and often competing theories of emotion processing: valence, RH, and approach/avoidance theories. With regards to the valence hypothesis, the RH does seem to identify negative emotions and the LH does not. Also, the LH does seem to identify positive emotions, and so those findings are not refuted in these pages, but it does not appear to be engaged in initial identification stages to indicate true comprehension of the emotional nature of the stimulus and it too frequently appears unable to do so. However, the LH does appear to have a propensity for positive emotional assessments at higher cognitive levels. There is no evidence that the LH ever adds a negative spin or interpretation. A strict dichotomous RH/negative and LH/positive division of labor has just not been supported, and I have cited many others who have drawn that same conclusion. The RH hypothesis, however, fits very nicely with this framework during early identification and comprehension of the emotional nature of a stimulus. It does appear as though the RH readily identifies and truly comprehends emotion of all valences, where this is not possible without an intact RH, and without the RH, even LH-dominant processes, such as verbal explanations and understanding appropriate emotion in social contexts are severely disrupted. However, that the RH entirely processes all aspects of emotion is not correct. As has been cited herein, the LH is important for regulation of negative emotion, so understanding and feeling emotion seems to be RH, but its modulation and attenuation may be LH. Kinsbourne and Bemporad (1984), Root et al. (2006), and Davidson (1984) also proposed an early role for the RH emotional identification and later involvement of the LH for response preparation.

Lastly, I have not directly incorporated much work on approach/avoidance into this paper because much of the theory hinges on the emotion of anger, and there are relatively few papers that include anger as compared to just happy and sad emotions. Moreover, as has been pointed out by Carver and Harmon-Jones (2009) and Tomarken and Zald (2009), by excluding anger, many investigators of the approach/avoidance hypothesis confound the two constructs by assuming positive always leads to approach and negative always leads to avoidance. The basic theory is that approach motivations are mediated by the LH, but avoidance/withdrawal motivations are mediated by the RH. As such, typical observations of asymmetries in emotion processing actually reflect motivation, not valence, asymmetries based on evidence that anger is negative, motivates approach and is also associated with the LH (Harmon-Jones, 2004; Harmon-Jones et al., 2013). While the approach/avoidance model has come under considerable criticism, there are enough solid findings to lend credence to the notion that LH has some involvement with the emotion of anger as an approach motivation (Harmon-Jones et al., 2009, 2011; Harmon-Jones and Harmon-Jones, 2010). However, anger does not appear to be as simple of an emotion as happiness or sadness. Anger can motivate two different classes of approach: amplification with aggressive behaviors (“kill them”) or attenuation and pro-social behaviors (“kill them with kindness”). Anger that motivates approach in the form of aggression [overt or covert as in leaning forward observed by Price and Harmon-Jones (2011)] has been extensively studied in humans and animals and will not be rehashed, here. Anger can also motivate avoidance, as in defensive aggression, “Your anger makes me afraid and want to run away or fight for my life,” or the more subtle “I’m so angry with you, I have to walk away.” Watson (2009) provided compelling evidence and reasoning indicating that anger is frequently associated with negative affect that motivates avoidance, whereas Harmon-Jones and colleagues have more thoroughly focused on approach aspects of anger. Whether anger motivates approach or withdrawal behaviors probably depends on the reward that can be attained from the resulting behaviors, even if the cause of the anger is unclear or experimentally manipulated. Reward likelihoods would be determined by contextual cues, social standing/dominance, and intensity of the stimulus. For example, an angry person may not aggress against their boss or someone of equal or higher social standing, but instead they may either approach and aggress against someone weaker or regulate the anger to create a more positive mood and responses. Conversely, an angry person may choose an avoidance strategy by running away if that is the safest path to goal attainment. For example, one may choose to avoid a spouse that caused their anger, because aggressing would jeopardize long-term and perhaps other, more important rewards. In either scenario, one could speculate approach and avoidance-motivated responses, depending on the social context, desired outcomes, and probably numerous personality tendencies. Zinner et al. (2008) also observed that social context may determine approach or avoidance motivations resulting from anger. As such, the construct of anger is more complex than other, basic emotions (also suggested by Schutter and Harmon-Jones, 2013), and simply assigning the entire construct of anger to a single hemisphere or motivational tendency is overly broad, and more importantly, not supported in the literature at this time. There are too few studies that investigate anger, its expression, interpretation, and resultant motivation in various contexts to populate an encompassing theory, but Harmon-Jones and colleagues are the only ones to have directly explored any aspect of anger associated with approach motivations in depth.

Interestingly, van Honk and Schutter (2007) have argued that anger associated with submissive/avoidant responses is associated with the RH, whereas anger associated with approach is LH. In the current framework, anger would be expected to be LH for more complex scenarios requiring a higher level thinking to incorporate emotional regulation, consideration of one’s social standing (perception by others), propositional knowledge (relationships among known entities), and verbalization. Conversely, anger would be RH under conditions of ambiguous or low-intensity threat, such as identifying facial expressions of complete strangers that appear on a screen (e.g., Jansari et al., 2000; Harciarek et al., 2006; Jackson et al., 2008; Balconi and Mazza, 2010; Bourne, 2011). Consider further that when anger is experimentally induced through insults, and the opportunity exists to aggress against the insulter (anger begetting greater anger), the LH is dominant in that approach-aggression (Hortensius et al., 2012), but that this context can also be interpreted as one of social dominance or standing. Extending this, Kelley et al. (2013) observed LH association with anger that results in aggression (approach) and greater RH association with anger resulting in rumination (avoidance). Rumination is interesting because it is not regulation (which I have argued to be associated with the LH), where rumination can lead to amplified aggression, but has also been associated with depression and anxiety (Pedersen et al., 2011). In the current framework, this could be interpreted as the RH maintaining the emotional experience until such a time as it could be acted upon by the LH for regulation, impression management, verbalization, and categorization. Findings of Harmon-Jones et al. (2003) also indicate that the LH becomes engaged when there is the possibility of alleviating an anger-inducing situation.

## Future Directions

First, the recent emotion literature is dominated by observations with clinical populations (neurologically intact and not) and electrophysiological techniques such as PET, fMRI, NIRS, rTMS, EEG, ERP. These studies, then, are all inherently noisy, with varying competencies and lesion locations of patients, and varying methods of interpreting and analyzing neurologic activation correlates. While neuroscientific contributions have been and will continue to be enormous, additional behavioral studies that utilize methodologies more complex than identification of laterally presented faces will enable a clearer understanding of the different competencies of the hemispheres during the normal course of emotion processing and generation of affective experience. For example, Hughes and Rutherford (2013) presented images from the International Affective Picture System (IAP, Lang et al., 2008) to the left or right visual fields as distractors while a central image was responded to by the non-distracted hemisphere. This technique could be applied to commonly used emotional stimuli, such as facial expressions. Moreover, an evolutionary approach to stimulus construction could also be utilized by varying familiarity with kinship or degree of drive reduction. The interaction between emotion and motivation is also proving to be interesting. The recent work of Spielberg, Heller and colleagues also provides interesting avenues for examining the integration of advanced emotional processing with motivation, approach, and avoidance dispositions, and have posited model of how different prefrontal areas interact to achieve goal-directed behaviors (e.g., Silton et al., 2010; Spielberg et al., 2011a,b; and Spielberg et al., 2013). Similarly, the work of Harmon-Jones and colleagues has presented some interesting and more complex methodologies by incorporating ostracism, cognitive dissonance paradigms, and experimentally inducing emotional states.

The proposed framework presents several avenues for exploration using simple emotional stimuli in non-clinical populations. For example, using signal detection analyses could help elucidate the true accuracy of the LH in judging emotions, and I have presented evidence for the prediction that the LH will show a high amount of “happy” false alarms and hits. Also, if both hemispheres independently process positive emotion, then a bilateral matching task (presenting two stimuli, one to each hemisphere) should not yield more response efficiency than unilateral presentations of the stimuli. If there is additive benefit to processing positive emotion, as suspected, then central presentation of an emotional stimulus should yield greater efficiency than left or right unilateral presentation. If the LH needs the RH to experience any negative emotion at all, then a bilateral matching task should yield greater efficiency than LH unilateral presentations, but perhaps less than RH unilateral presentations if the RH can handle it within hemisphere. The few matching tasks that have been reported warrant replication.

Further, to investigate a cross-hemispheric transfer of emotional information, one hypothesis could be that priming the RH with valenced information will bias verbal descriptions of neutral stimuli, whereas priming the LH with valenced information may have no effect at all on RH interpretation of neutral stimuli. This would be akin to “tricking” the LH, as discussed by Baynes and Gazzaniga (2000). Alternatively, unilateral emotional primes could be followed by ipsilateral or contralateral ambiguous targets. Such stimuli can be faces (as in the Emotional Hexagon Test used by Bate et al., 2013), objects and situations (as in the International Affective Picture System), complex social interactions (a breakup), or words. One could also “trick” the LH in its own territory: language. For example, sentences that have many positive words but convey a negative message (“I love the way you are so fantastically oblivious to your surroundings”) or vise versa (“You make me sick with how bad you are at lying”) may confuse the LH, resulting in a generally positive interpretation, especially if presented simultaneously with a congruous or incongruous context. Applying the unilateral distraction technique of Hughes and Rutherford would enable study of each hemisphere’s contribution in non-clinical populations. For example, GSR for each hand could be measured under conditions of unilateral distraction. If the LH responds to negative information only after it has been shared by the RH, then distracting the LH while presenting a negative stimulus to the RH, followed by an undistracted neutral trial should result in no LH bias as compared to trials where the LH is not distracted at all. The time course of inter-hemispheric sharing can be explored by observing the activity of structures within the DMN during and after presentation of emotional stimuli at varying durations of central and lateralized exposure (e.g., from 25 through 200 ms). Response requirements can also be varied after different stimulus onsets to investigate the stage of processing for emotional information. For example, identifying emotional versus neutral faces should take place early (RH), identifying emotional words should be later (bilateral or LH), and determining if someone gave an appropriate or alternate social responses may be even later (LH). This, combined with unilateral presentations would be useful in detangling the precise contributions of each hemisphere to different aspects of emotional processing. Following the lead of Compton et al.’s (2005) matching task, one could also help determine the depth of each hemisphere’s contribution by similarly presenting different emotional expressions to each hemisphere, followed by a unilateral conceptual match, rather than a perceptual match, if the target is a different person from either samples or a congruent/incongruent picture from the IAP.

One last avenue that would be promising for investigating hemispheric collaboration in emotion processing is handedness. Degree of handedness is the extent to which individuals use one hand for performing a series of tasks. Consistent-handers usually or always prefer use of the same hand (either left or right) whereas inconsistent-handers are more varied in their hand preferences, using the left for some tasks, right for others, or having no hand preference. Consistent and inconsistent-handers show reliable differences on behavioral tasks that suggest greater independence of the hemispheres in consistent-handers and greater collaboration in inconsistent-handers. As such, consistent-handers have an advantage over inconsistent-handers on tasks that require hemispheric independence, but inconsistent-handers have the advantage when tasks require a collaborative or cross-hemispheric strategy (Christman, 2001; Propper et al., 2005; Lyle and Martin, 2010). Inconsistent-handers may also have an advantage for tasks that are predominately RH, and Propper et al. (2010) observed that inconsistent-handers rate their subjective mood more negatively than consistent-handers. Consistent with the emotion literature and the hypothesis that negative emotions are distributed across the hemispheres for different kinds of processing, then inconsistent-handers are predicted to have an advantage for the identification and regulation of negative emotion. Niebauer (2004) reported that consistent-handers are more likely to ruminate and inconsistent-handers are more likely to self-reflect. He argued that inconsistent-handers are self-reflective because RH experiences update the LH fringe of consciousness (sense of rightness and general feelings). The increased inter-hemispheric communication of inconsistent-handers facilitates this process, whereas decreased inter-hemispheric communication limits this process, resulting in ruminating consistent-handers. Applying Niebauer’s reasoning to emotion, it is predicted that inconsistent-handers may be better at regulating emotions than are consistent-handers, because emotional regulation may require cross-hemispheric collaboration. Relatedly, Jasper et al. (2008) observed that inconsistent-handers are better at generating alternative outcomes to stories than are consistent-handers, and the reappraisal strategy for emotional regulation requires generating alternatives to real-life events. Indeed, inconsistent-handers should show an advantage for any aspect of emotional processing that requires hemispheric collaboration, such as including more emotional content in verbal descriptions of scenes and events. Conversely, inconsistent-handers may have a disadvantage for emotional processing that is better accomplished by the LH alone or by the hemispheres, independently. For example, inconsistent-handers may have more difficulty than consistent-handers ignoring a correct emotional experience or identification in favor of a popular or socially acceptable one. Inconsistent-handers may show processing advantages for identification of negative emotions and they may also have more difficulty separating the genuine experience of the RH from the LH idealized experience.

There have been very few papers directly investigating emotion processing by degree of handedness. One paper suggests that inconsistent-handers may experience emotion more genuinely than consistent-handers. Farina et al. (2012) report that inconsistent-handers show greater emotional distress than do consistent-handers to a traumatic event (earthquake). In this instance, an earthquake truly is traumatic, and experience of associated negative emotion is expected. Because the RH appears to be the on-line information processor of real-world emotional stimuli, inconsistent-handers may have a greater understanding (ability to process, feel) of the traumatic event. Consistent-handers however, may have less of a connection to the RH emotion and so display less distress. Another study by Mikkelson et al. (2006) examined consistent and inconsistent hander differences in expressiveness and emotional control. While these authors report differences as having been observed (opposite to what is predicted, here), they do not report the statistical inferences that led to their conclusions. Certainly, more research will enable stronger speculations.

Two other papers, using different methodologies have seemingly opposing conclusions. For example, Bourne (2008), using a free vision chimeric happy-face task, observed that degree of handedness is related to degree of RH lateralization for both positive and negative emotions. This supports the RH hypothesis for consistent-handers, but may also suggest that an increased collaboration between hemispheres of inconsistent-handers results in no asymmetries for perceptual identification of happy faces. This is also predictable by the current model, where either hemisphere can identify happy emotions, consistent-handers lateralizing the task to one hemisphere and inconsistent-handers using either hemisphere. Brunyé et al. (2012) observed stronger left and right spatial biases for remembering positively and negatively valenced stimulus locations for consistent-handers. This could be used to support the valence hypothesis. While these do appear to be conflicting, their different methodologies and responses could also reflect different stages or types of emotional processing, Bourne for valence identification, and Brunyé et al. for more complex associations. Both do suggest that inconsistent-handers are less lateralized for emotional stimuli. Using degrees of handedness to research the extent to which the cerebral hemispheres interact in the processing of emotion is a largely unexplored, but promising territory. Moreover, the experimental manipulation of bilateral eye movements has been demonstrated to increase inter-hemispheric processing (Christman et al., 2003; Parker and Dagnall, 2010) particularly in consistent-handers (Lyle et al., 2008; Shobe et al., 2009). The use of degree of handedness and bilateral eye movements are two methods that can be used to explore inter-hemispheric collaboration during various types and phases of emotional processing.

## Summary

- The RH pulvinar–amygdala–superior colliculus pathway is a fast, coarse, and unconscious processor of emotional stimuli. The cortico-pulvinar–cortical pathway serves to orient and direct cortical attention to emotional stimuli, engaging the DMN for higher order emotional processing. Pulvinar nuclei of the thalamus may be critical for synchronizing cortical neurons with RH subcortical processing of emotion. Processing negatively valenced stimuli, in particular, results in inter-hemispheric synchrony between the structures of the DMN, suggesting sharing of negatively valenced information across the hemispheres.
- Commissurotomized and AgCC patients show deficits identifying emotions, particularly regarding negative emotions. They are also impaired at linking emotional experiences with verbal descriptions. The intact RH and LH of these patients suggests normal intra-hemispheric abilities (e.g., speech, recognizing idiom meaning, physiological arousal to emotional stimuli), and so their emotional deficits are likely due to the inability of the RH to share emotional information with the LH.
- Left hemisphere activity has been associated with regulation of negative emotions by attenuating emotional experience, avoiding emotional distractions, reinterpreting events as positive or benign, or by having an advantage at problem solving strategies used for emotional regulation. Patients with PD and depression have difficulties with emotional regulation and also may have LH hypoactivity. RH hypoactivity associated with mania may be due to the absence of RH input. Mania does not appear to be due to a LH that can only process and affect positive emotions because AgCC and commissurotomized patients do not show manic-type speech or positive content discourse. The role of the LH in emotional regulation and the exhibition of mania in the absence of RH activity suggest that the LH is detached from feeling associated with, and comprehension of emotional stimuli and situations.
- Evidence from a case study and signal detection analysis suggests that the LH has default bias to respond positively. The LH also has a tendency to identify positive traits in ourselves, generating an idealized sense of self that enables impression management. This LH tendency extends to the perception of others. A consistent positive perception of ourselves and others has evolutionary advantages by promoting social interaction.
- The LH transforms real-world, event-specific emotions of the RH into propositional knowledge and verbal codes. The LH remembers gist information about positive valence stimuli, interprets linguistic aspects of prosody, and like AgCC patients, uses evaluative and concrete words rather than feelings to describe personal emotional events. The LH appears to parse emotion stimuli and subjective affect into linguistic elements, semantic meaning, and verbal labels. LH brain structures activated during emotion processing are language-related.
- Both the LH and the RH can identify positive emotions independently, as there is within-hemisphere activity for unilateral presentations, unilateral LH damage does not always produce positive valence deficits, and there is consistent evidence for a RH/left side bias in processing positive emotions. There is ample evidence to suggest that the LH does not identify, comprehend, or experience negative affect. Instead, emotional aspects of negative valence stimuli are processed by the RH, only. In the normal brain, where bilateral activity is observed during processing of negative valence, the LH must use information received from the RH for verbal and propositional contributions to emotion processing, and emotional regulation.
- The currently proposed theory suggests that the LH contribution to emotion is not at a basic, comprehension level. Instead, the LH receives this type of information from the RH and integrates it into higher level cognitions. As such, the LH and RH contribute to different components or levels of emotion processing. There are many methodologies that have been under-utilized in the study of hemispheric asymmetries of emotion that have been successfully utilized in other areas. As such, to date, we have little understanding of the degree to which each hemisphere processes emotional information or their relative contribution to the many aspects of the emotional experience.

## Conflict of Interest Statement

The author declares that the research was conducted in the absence of any commercial or financial relationships that could be construed as a potential conflict of interest.
